# Genetic diversity of the Thao people of Taiwan using Y-chromosome, mitochondrial DNA and HLA gene systems

**DOI:** 10.1186/s12862-019-1389-0

**Published:** 2019-02-27

**Authors:** Jean A. Trejaut, Frank Muyard, Ying-Hui Lai, Lan-Rong Chen, Zong-Sian Chen, Jun-Hun Loo, Jin-Yuan Huang, Marie Lin

**Affiliations:** 1 Molecular Anthropology and Transfusion Medicine Research Laboratory, Mackay Memorial Hospital, Taipei, Taiwan; 2 Department of French Studies, National Central University, Taoyuan Taiwan & French School of Asian Studies (EFEO), Taoyuan, Taiwan

**Keywords:** Phylotree, Human population genetics, Mitochondrial DNA, Thao Taiwan aboriginal people, Phylogeography

## Abstract

**Background:**

Despite attempts in retracing the history of the Thao people in Taiwan using folktales, linguistics, physical anthropology, and ethnic studies, their history remains incomplete. The heritage of Thao has been associated with the Pazeh Western plains peoples and several other mountain peoples of Taiwan. In the last 400 years, their culture and genetic profile have been reshaped by East Asian migrants. They were displaced by the Japanese and the construction of a dam and almost faced extinction.

In this paper, genetic information from mitochondrial DNA (mtDNA), Histoleucocyte antigens (HLA), and the non-recombining Y chromosome of 30 Thao individuals are compared to 836 other Taiwan Mountain and Plains Aborigines (TwrIP & TwPp), 384 Non-Aboriginal Taiwanese (non-TwA) and 149 Continental East Asians.

**Results:**

The phylogeographic analyses of mtDNA haplogroups F4b and B4b1a2 indicated gene flow between Thao, Bunun, and Tsou, and suggested a common ancestry from 10,000 to 3000 years ago. A claim of close contact with the heavily Sinicized Pazeh of the plains was not rejected and suggests that the plains and mountain peoples most likely shared the same Austronesian agriculturist gene pool in the Neolithic.

**Conclusions:**

Having been moving repeatedly since their arrival in Taiwan between 6000 and 4500 years ago, the Thao finally settled in the central mountain range. They represent the last plains people whose strong bonds with their original culture allowed them to preserve their genetic heritage, despite significant gene flow from the mainland of Asia.

Representing a considerable contribution to the genealogical history of the Thao people, the findings of this study bear on ongoing anthropological and linguistic debates on their origin.

**Electronic supplementary material:**

The online version of this article (10.1186/s12862-019-1389-0) contains supplementary material, which is available to authorized users.

## Background

Taiwan’s multicultural and multilingual population reached 23.5 million in 2016 [1]. Mandarin, the official language, is almost universally used and understood, while significant portions of the population speak other Sinitic languages, such as Minnan and Hakka groups originally from Southeast China. It is believed that the very first fully modern humans arrived on the island between 20,000 and 30,000 years before present (YBP) in very small numbers during the late Pleistocene when Taiwan was still a part of the East Asian mainland [2]. Although a few traces of this era can be inferred from the genetic profile of the current population [3–6], and from archeological artifacts of Paleolithic cultures [2, 7], it is believed that Palaeolithic groups disappeared during the Last Glacial Period of the Mesolithic Age, or at the latest, around the time the Neolithic groups arrived in Taiwan [2, 7–9], and their genetic identity, origin, and continuity with the extant aboriginal populations of Taiwan remains unresolved.

Today there are 16 groups of officially recognized indigenous peoples in Taiwan (TwrIP) who represent approximately 2.2% of the Taiwan population. These groups speak Austronesian languages. The greatest genealogical diversity of the Austronesian languages is found in Taiwan, where they diversified and expanded from the ancestral Proto-Austronesian languages arriving from the East Asian Mainland 6000 YBP [7, 10] with the Neolithic colonization of the island. This language group most likely reached its present diversity at the beginning of the Neolithic era, and are often referred to as the Formosan languages. Subsequent human entries include at least Metal Age Austronesian groups from Southeast Asia, European, Chinese, Japanese colonial settlers, and post Second World War Chinese exilés, each with substantial cultural and genetic impacts on the island’s population [5, 11–13].

**Fig. 1:**
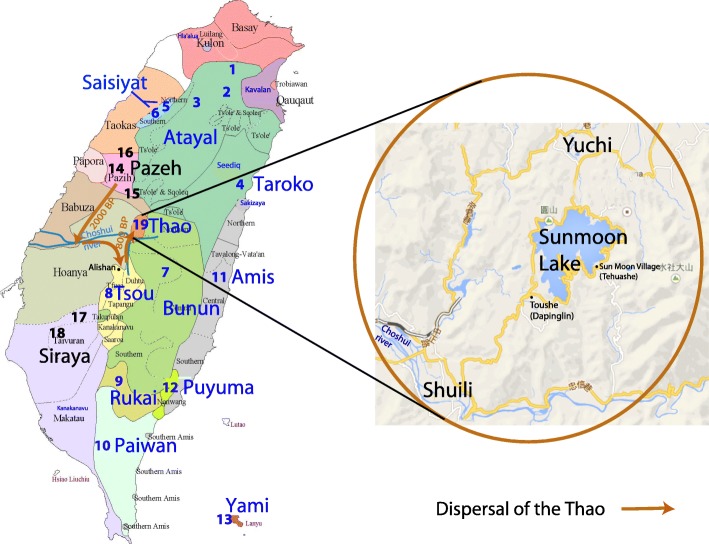
Geographic distribution of the Taiwan indigenous peoples. Numbers indicate the sampling locations of the people: Atayal (1-Wulai, 2-Chenshih, 3-Wufen); Taroko (4-Hsiulin); Saisiyat (5-Wufen, 6-Nanchuang); Bunun (7-Hsin-I); Tsou (8-Tapang); Rukai (9-Wutai); Paiwan (10-Lai-I); Amis (11-Kuangfu,); Puyuma (12-Peinan); Tao (13-Lanyu); Pazeh (14-Fengyuan, 15-Puli,16-Liyutan); Siraya (17-Tanei, 18-Tsochen) and the Thao people (19) scattered from Yuchih/Yuchi Village to Shueili/Shuili Village in Nantou County with about 600 Thao people today

A full list of the recognized indigenous peoples of Taiwan (TwrIP), as well as some of the more commonly cited unrecognized tribal groups includes the groups recognized by the Taiwan government: Amis, Atayal, Bunun, Hla’alua, Kanakanavu, Kavalan, Paiwan, Puyuma, Rukai, Saisiyat, Tao (or Yami), Tsou, Taroko, Sakizaya, Seediq, and Thao. Other groups such as the Babuza, Basay, Hoanya, Ketagalan, Luilang, Makatao, Pazeh/Kaxabu, Papora, Qauqaut, Siraya, Taokas, and Trobiawan groups, largely Taiwan plains peoples, are known collectively as the Pingpu (TwPp) and are not recognized by the government. They represent 0.5% of the Taiwan population, their languages are extinct or nearly so, and all speak Mandarin or other Sinitic languages. Most TwrIP today live in the Central Mountain Ranges or on the East coast of Taiwan, except for the Yami, who inhabit Orchid Island (Lanyu) southeast of Taiwan. Each group has its own Austronesian language. Among the 500,000 Taiwan indigenous people, the Thao, with just over 300 individuals at the time of sampling represents the smallest group [1]. Presently reaching 660 dispersed members, approximately 300 people speak the original language at a very poor level, and with only 15 competent speakers, their language is close to extinction [14, 15]. The Thao now live in the central mountain range (Fig. 1), but phonological and lexical evidences suggest that they are more closely related to western plains-dwelling cultures such as the Pazeh [7, 16]. It has been suggested that they must have interacted with ancestral groups of the plains peoples while living along the Choshui river in south-central Taiwan long before moving eastward to the central mountain ranges approximately 2000 years ago [7, 15–17]. It has also been suggested that Thao moved to the Sun Moon Lake area approximately 800 years ago from an initial settlement further south [15] near Alishan (Fig. 1) in close proximity to the Tsou people. It is possible that they moved there during the Qing Dynasty (1644–1912), at the end of the eighteenth century, when the practice of tenant farming by the new East Asian settlers led to draining of the farmlands, forcing the Thao to abandon their traditional plains dwellings and retreat to the hills [17].

The Thao comprised three major clans, the Yuan, Shi, and Mau clans. The arrival of the Han from China over the last four centuries bringing armed conflicts and infectious diseases reduced the population of the plains and mountain peoples and brought the Thao people, who were already small in number, to the brink of extinction [18].

During the period of Japanese colonial administration (1895–1945), the Japanese government began to modernize Taiwan. In 1919, the colonial authorities decided to build a dam on Sun Moon Lake. Most Thao inhabiting the area were forced to relocate to nearby areas [19]. Further, the Chi-Chi earthquake of 1999 damaged or destroyed 80% of the houses of the Thao people and sent many to look for employment in other cities.

After many episodes of displacements and regrouping, the Mau clan now lives in Shuili and Dapinglin (presently Toushe or Puzi) villages, south of Sun Moon Lake, and part of the Shi clan who previously resided further north in Yuchi have now rejoined the groups in Tehuashe (presently Sun Moon village east of Sun Moon Lake) [20].

However, the home of the Thao clans before they reached the Sun Moon Lake region remains unclear. Were they really in contact with the Pazeh people on the western plain and later came up along the Choshui river [16]? Did they temporarily settle in the neighborhood of the Tsou people [15]? A 1921 tourist industry version of a tribal legend of the chasing of a white deer that finally lead the Thao to Sun Moon Lake may indicate that the Thao came from further south, possibly the Alishan region near the current home of the Tsou. Interestingly, in 1951, according to this account and following an initial Japanese anthropological classification allowing recognition of only a limited number of Taiwan groups, the Tsou and Thao were classified as belonging to a single group: the Tsou People [20, 21]. However, this classification, along with the origin of the Thao, remains under debate.

Further anthropological studies showed that the Thao peoples were very different from the Tsou, and although, like the Tsou, Thao peoples lived by farming, hunting, fishing, and collecting, and now principally sell artifacts to tourists they still venerate their ancestral spirits and have conserved a rich and unique culture that is different from the Tsou [21] or other neighboring peoples. More importantly, the Thao people have unique rituals, such as rhythmic pestle music and tooth pulling, and scholars nowadays describe them as a unique socio-cultural group [21]. The Thao people are a localized kin group of patrilineal exogamous descent. Traditionally, a single hereditary clan maintained control of the leadership whereby the chief, who made decisions about ceremonial rituals, had this authority passed from his father and if there was no first-born son, then the next male kin would inherit the title [17]. Information appertaining to specific clans is not included in this study. All Thao now live in the region to the south of the Atayal and Saisiyat peoples and are close neighbors to the Bunun in the southeast with whom they share some similar linguistic and social traits.

Morphometric differences presented by Yu Chin-Chuan and Tseng Tsung-Ming [9] were coupled with the geographic distribution of other TwrIP. These included 13 items of observation, 20 morphometric measurements and 20 indexes calculated from these measurements [9]. In brief, the physical characteristics of most Formosan aborigines have been described as 1. straight hair with very little wavy hair, 2. black hair with some black-brown, 3. Brown or dark-brown eye, 4. a high percentage of double-eyelids, 90 to 100%, and 5. Mongoloid folds 61 to 90%. The Thao showed no significant difference from other TwrIP except that they have a lower percentage of Mongoloid folds. Further, Yu and Cheng’s results show that the Thao are physically more similar to the Bunun, the Atayal, and to the Paiwan, and were more distant from the Amis further to the east and the Yami. Intriguingly, the same study also described physical anthropological traits closer to the Hakka, perhaps suggesting gene admixture between Thao and non-Aboriginal groups and/or drift.

The official classification of ethnic groups today considers the individuals or groups’ history, their self-perception, the government’s perception, and the findings of researchers in various fields such as linguistics, culture, and ethnology [21, 22]. Past or present acculturation in Taiwan, sinicization, and recent advances in technology have also influenced the way people view themselves, each other and where they prefer to live. Presently, the impact of genetics on all fields of study [23] and its easy availability to the public and scientific communities have become generally well accepted, better understood, and taken very seriously. By ascertaining the magnitude and spatial distribution of the genetic diversity in Taiwan, our study aims to shed greater light on the genetic heritage of the Thao people and to detect evidence of past admixture between regional groups. For this, we carried out analysis of the polymorphism of paternally inherited non-recombining Y chromosome (NRY), of the maternally inherited mitochondrial DNA (mtDNA), and of the diploid human leukocyte antigens (HLA-A, −B and -DRB1) among individuals from most groups and locations within Taiwan, the Philippines, and Fujian.

## Results

### Genetic diversity

The ranges of genetic diversity in the Taiwan Austronesian speaking groups (Table 1) seen across the HLA-A, −B and -DRB1 loci (mean range 0.634 to 0.813), the HLA-A-B-DRB1 haplotypes (0.875 to 0.979) and mtDNA loci (0.730 to 0.965) were generally lower than seen in Taiwan Sinitic speaking groups, Fujian, non-TwA, and TwPp (HLA alleles: 0.833 to 894; HLA haplotypes: 0.976 to 1.000 and mtDNA: 0.977 to 0.990) (Table 1). Across the Y-SNP loci, the difference in gene diversity between groups was more pronounced. It first separated the non-TwA and TwPp groups (Y-SNP 0.689 to 0.889 and Y-STR 0.941 to 0.999) from the Southern TwrIP (Y-SNP 0.461 to 0.701 and Y-STR 0.834 to 0.968), and even further from the Thao, the Tsou and the northern indigenous peoples (Y-SNP 0.095 to 0.229 and Y-STR 0.318to 0.775). Further, while the average number of HLA alleles [24, 25] and mtDNA haplogroups observed among mainland Asians, non-TwA and TwPp (Additional file 1: Table S1) were fairly high, the number of Y-SNP haplogroups seen among TwrIP did not reach values greater than four (k ≤ 4). Finally, tests of neutrality for Thao, Tajima *D* (*D* = -0.53; *p* > 010) and the more powerful Fu’s *Fs* test (*Fs =* 1.46; *p* > 0.75) did not indicate a departure from neutrality expectation and were in range with most values observed among other TwrIP groups (Additional file 2: Table S9).

**Table 1:** Gene Diversity in three gene systems (NRY, HLA, and mtDNA)

Populations				Gene Diversity				
				Y chromosome		Mitochondrial DNA	HLA A,B and DRB1	
				Y-SNP haplogroups ± SD	Y-STR haplotypes ± SD (7 STRs)	mtDNA Haplogroups ± SD	Aleles ± SD	Haplotypes ± SD
Sinitic speakers	East China	Fujian		0.849 ± 0.017	0.979 ± 0.006	0.990 ± 0.001	0.894 ± 0.022	0.997 ± 0.003
	Taiwan sinitic speaking groups	Taiwan mixed sample		0.887 ± 0.007	0.999 ± 0.000	0.990 ± 0.000	0.879 ± 0.022	0.992 ± 0.001
		Hakka		0.889 ± 0.020	0.985 ± 0.006	0.987 ± 0.002	0.892 ± 0.030	1.000 ± 0.002
		Minnan		0.886 ± 0.013	0.987 ± 0.006	0.990 ± 0.001	0.893 ± 0.016	0.996 ± 0.002
		All above		0.893 ± 0.006	0.983 ± 0.003	0.990 ± 0.001	0.882 ± 0.021	0.995 ± 0.001
		Pazeh (Pingpu)		0.689 ± 0.020	0.941 ± 0.014	0.977 ± 0.006	0.878 ± 0.023	0.990 ± 0.005
		Other Taiwan Plain tribes (Pingpu)		0.859 ± 0.008	0.994 ± 0.001	0.981 ± 0.001	0.833 ± 0.030	0.976 ± 0.004
Austronesian speakers	Taiwan Austronesian speaking groups (Formosan)	Northern Tribes	Atayal	0.177 ± 0.049	0.518 ± 0.060	0.886 ± 0.012	0.809 ± 0.044	0.966 ± 0.004
			Taroko	0.095 ± 0.062	0.447 ± 0.096	0.730 ± 0.025	0.813 ± 0.038	0.976 ± 0.004
			Saisiyat	0.229 ± 0.080	0.775 ± 0.039	0.869 ± 0.014	0.728 ± 0.064	0.958 ± 0.008
		Central tribes	Thao	0.227 ± 0.095	0.351 ± 0.010	0.894 ± 0.023	0.788 ± 0.058	0.939 ± 0.017
			Bunun	0.490 ± 0.025	0.886 ± 0.012	0.893 ± 0.008	0.768 ± 0.056	0.945 ± 0.014
			Tsou	0.181 ± 0.056	0.318 ± 0.014	0.920 ± 0.010	0.692 ± 0.078	0.918 ± 0.015
		Southern Tribes	Amis	0.669 ± 0.025	0.909 ± 0.024	0.910 ± 0.013	0.702 ± 0.067	0.909 ± 0.010
			Rukai	0.461 ± 0.060	0.905 ± 0.021	0.905 ± 0.012	0.686 ± 0.079	0.945 ± 0.014
			Paiwan	0.701 ± 0.029	0.909 ± 0.024	0.929 ± 0.005	0.634 ± 0.076	0.917 ± 0.017
			Puyuma	0.688 ± 0.048	0.968 ± 0.009	0.944 ± 0.005	0.785 ± 0.058	0.977 ± 0.006
			Yami	0.627 ± 0.040	0.834 ± 0.023	0.852 ± 0.009	0.711 ± 0.063	0.875 ± 0.019
		All TwMtA (no Thao)		0.603 ± 0.018	0.831 ± 0.017	0.965 ± 0.001	0.797 ± 0.010	0.979 ± 0.001
	Malayo-polynesian speaking groups	Batan (Ivatan)		0.726 ± 0.039	0.935 ± 0.018	0.923 ± 0.012	0.821 ± 0.037	0.963 ± 0.012
		Philippines(no Batan)		0.893 ± 0.007	0.935 ± 0.018	0.952 ± 0.003	0.833 ± 0.023	0.998 ± 0.003

### Non-recombining Y chromosome (NRY) of the Thao

**Fig. 2:**
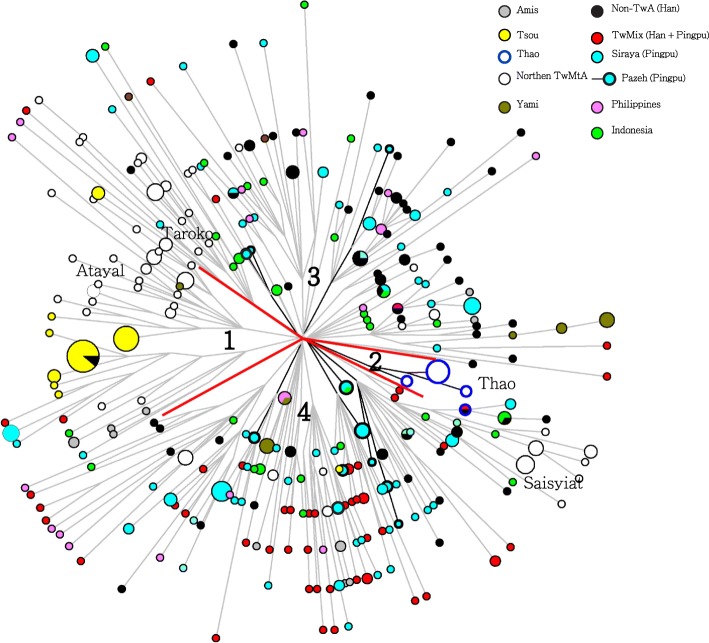
Reduced Joining Network of haplogroup O1a1*-P203 constructed using 17 Y-STR loci. Haplogroup O1a1*-P203 is prominent among Thao (87.5%) and the Taiwan northern peoples Tsou, Bunun, and Saisiyat. Color codes: white = Northern Taiwan aboriginals (Atayal, Taroko, Saisiyat), red = Southern Taiwan Aboriginals (Rukai, Paiwan, Puyuma), yellow = Tsou, light blue = Taiwan plains peoples/Pingpu peoples, black = non-TwA (Fujian and Taiwan Han), pink = Filipinos, and green = Indonesia. Circles are sized proportional to the frequency of the Y-STR haplotypes and branch lengths are proportional to the number of mutational steps. Marked quadrants (1 to 4) delineate four (non-restricted) sub-networks of O1a1*-P203 (1: Taiwan Northern groups, 2: Thao, 3: non-Taiwan Aborigines and 4: Taiwan Plain peoples/Pingpu peoples and Southern peoples). The gray crossed nodes with a blue circle in sector 2 represent Thao

All Y-SNP haplogroups observed in the Thao sample (16 males out of 30 individuals) were para-groups of macro-haplogroup O1; namely, O1a*-M119 (*n* = 1), O1a2-M50 (n = 1) and O1a1*-P203 (*n* = 14, 87.5%) (Additional file 1: Table S1). These results corroborate a previous report [26] where 81.8% of Thao males belonged to haplogroup O1a while the remainder of the data set showed little presence of haplogroups K, O1a2, or O3. Li’s dataset [26] was not included in our analysis because of their differing definitions. They used a lower Y-SNP definition that did not allow clear assignation of haplogroup O1a1*-P203, and they used only five Y-STRs compared to 16 in our panel. With the exception of Bunun, who showed a predominance of haplogroup O1a2-M50 and the highest frequency of O2a1a-M88 seen in ISEA [27], the Thao Y-SNP profile was similar to that of other TwrIP, particularly the Atayal, Taroko, Saisiyat, and Tsou who, together, share the highest occurrence of O1a1*-P203 in the world (87.5 to 95%) (Fig. 4 and Additional file 1: Table S1). In the Y-STR Median-Joining network (Fig. 2) of haplogroup O1a1*-P203, comprising data from the Philippines, Indonesia, and all Taiwan ethnic groups, the diversity of the Y-STR haplotypes clearly suggested the existence of several sublineages of O1a1*-P203 and placed Thao into a separate TwrIP cluster distinct from all other TwrIP groups, the Philippines, and Indonesia. Further, the molecular variation of haplogroup O1a1*-P203 (Table 2), estimated from Y-STRs and the rho statistic [28], produced results similar for Thao and Tsou (1590 ± 690 years and 2182 ± 1816 YBP respectively) (Table 2).

**Table 2:** Molecular age estimates of subtypes of haplogroup O1 in Thao and other groups using seven Y-STRs

Y Haplogroups		Taiwan					Philippines		Western Indonesia *N* = 192
		Taiwan Han *N* = 446	Pingpu		TwrIP				
			Pingpu (no Pazeh) *N* = 370	Pazeh *N* = 44	TwrIP (no Thao) *N* = 339	Thao *N* = 16	Batan *N* = 24	Philippines (no Batan) *N* = 146	
O1a*-M119	ya (n)	16,560 (5)	13,380 (7)	na	19,960 (28)	na	10,820 (10)	20,360 (15)	14,590 (11)
	± SE	± 4390	± 2575		± 5530		± 2850	± 5650	± 5560
O1a1*-P203	ya (n)	8590 (50)	10,740 (93)	5669 (21)	16,290 (210)	1590 (14)	9830 (10)	16,050 (15)	7280 (32)
	± SE	± 3270	± 6600	± 2628	± 5880	± 689	± 3510	± 3570	± 4.00
O1a2-M50	ya (n)	na	8630 (30)	3106 (5)	17,540 (67)	na	na	12,480 (16)	7060 (11)
	± SE		± 3180	± 1464	± 3560			± 5950	± 2630

*ya* thousand years ago, *TwrIP* Taiwan recognized Indigenous People, *PingPu* Taiwan western Plain tribes (Not including Pazeh)

n: number of individuals bearing this haplogroup in the ethnic group

na: age was not estimated if less than 5 individuals

Han include Minnan, Hakka and a Taiwan Mixed group (AD)

N=Population size

The seven Y-STRs used were: DYS19, DYS389 I, DYS389 II DYS390, DYS391, DYS392, and DYS393

### Mitochondrial DNA

We distinguished eight different mtDNA haplogroups among the Thao people. All fell within the mtDNA paragroups B4, B5, E1a1, F1a, F4b1, and M8a2’3 (Fig. 4, Additional file 1: Table S1 and Additional file 3: Supplementary text 1). While all the clades had an ancestral origin in southeastern mainland Asia, only two, F1a’ and M8a2’3′ were shared with Fujian. Members of the B4b1 clade have been identified across the East Asian mainland, in Japan, and among the Negrito groups of the Philippines [29–31]. They are thought to have reached these regions prior to the Out of Taiwan (OOT) dispersal 4000 YBP [30, 32, 33]. Haplogroup subtypes B5a2a2b, B4b1a2f3, B4b1a2g, B4b1a2k, and F4b1c’d accounted for 63.3% of the Thao mtDNA gene pool (Additional file 1: Table S1, Additional file 4: Table S2, Additional file 5: Table S3, Additional file 6: Table S4 and Additional file 7: Table S5). They were commonly seen among the northern and central TwrIP, and are unique to Taiwan. The presence of different subtypes of B4b1a2 in the Philipines (Additional file 4: Table S2) suggests separate expansions of the B4b1a2 clade in Taiwan and the Philippines between 5400 and 9700 YBP [30] (Table 3).

**Table 3:** mtDNA molecular variation (age) using rho total (Soares et a. 2009)

mt Haplogroups	Age	
B4b1a2 (np T6216C)	9314 ya	CI 6958–11,032
B4b1a2f (nps G709A, T14110C)	4951 ya	CI 1248–8733
B4b1a2f2 (nps G709A, T14110C, A10313c)	4343 ya	CI 2442–6268
B4b1a2f3 (nps G709A, T14110C, G6260A)	2585 ya	CI 1922–3551
B4b1a2g (np C16365T)	4225 ya	CI 660–7873
B4b1a2k (np G207A!, A8014G, C16400T)	4687 ya	CI 2423–76,384
B4c1b2a2 (nps T146C, T8772C)	5881 ya	CI 0–13,399
B5a2a (nps A93G, G11149A, C14149T)	27,943 ya	CI 14728–42,395
B5a2a2 (np T8614C)	13,426 ya	CI 6631–20,474
B5a2a2b (nps C5027T, C8059T)	6228 ya	CI 2760–9772
B5a2a2b1 (np A4824G)	4264 ya	CI 1042–7554
E1a1anew (nps T6620C, C14766T, G16129A)	na	
F1a3a3 (np C15452T)	4290 ya	CI 563–8110
F1anew (nps G6962A, T10604C, A14053G)	4343 ya	CI 513–8270
F4b1 (np A10097c)	4317 ya	CI 1296–7280
F4b1c (nps 8548 s 14215 s 15924 s)	1287 ya	CI 0–3071
F4b1d (np G513A)	513 ya	CI 10–1018
M8a2’3′ (np C16184T)	na	

ya: thousand years ago

np: nucleotide position

### HLA

HLA characterized clear genetic differences between the Continental East Asian multilinguistic areas, such as Fujian, the non-aboriginal or mixed groups (Minnan, Hakka, and TwPp), and the Austronesian speaking TwrIP (Fig. 4). In brief, excluding HLA-DRB1*08:02 (1.67%) and DRB1*13:12 (1.67%) (Additional file 1: Table S1), all other Thao HLA-A, B, and DRB1 alleles were seen at various frequencies in most other Austronesian and non-Austronesian speaking groups of Taiwan and Southeast China [34–36]. Among these groups, the sole difference in this apparent homogeneity of distribution observable within the groups was most likely brought about by drift. By contrast, except for those haplotypes conserved by selection, recombinations between HLA loci contribute to greater HLA haplotype diversity. Accordingly, we used the Expectation Maximum likelihood procedure in Arlequin 3.5.2.2 to infer HLA-A-B-DRB1 haplotypes and use them as indicators to retrace the events of past migrations and the dispersal history of all groups studied [37, 38]. For example, according to Chu et al. (2004) and Lin et al. (2001) the profile of the distribution of characteristic bi-loci haplotypes seen in Thao and TwrIP (HLA-A*02:07-B*4601, A*11:01-B*15:01:01, A*11:01-B*40:01, A*11:01-B*55:02, A*33:03-B*58:01, and B*58:01-DRB1*03:01:01) is significantly different from the profile seen in non-TwA [34, 36]. Here, using tri-loci haplotypes, only six (26%) of the 23 Thao triplet haplotypes (Fig. 4 right, Additional file 1: Table S1, and S8) were shared between the Thao (k = 23 haplotypes) and Fujian (k = 82 haplotypes) out of 962 haplotypes in the complete data set. This pattern remained consistent when analyzing other TwrIP groups. In addition, while three HLA haplotypes represented 55% of the Thao profile, HLA-A*24:02-B*40:01-DRB*11:01, HLA-A*24:02-B*39:01-DR*08:02, and HLA-A*24:02-B*13:01-DR*12:02, the MDS plot located the Thao among the central Taiwan mountain peoples, and two closely related southern aboriginal peoples, the Paiwan and Rukai (Fig. 4).

Last, the exact test of the Hardy-Weinberg Equilibrium of Thao obtained from all HLA loci using a 100,000 Markov chain length [39] did not show a departure from expectations (*p* > 0.12) and corroborated the results described above for mtDNA (data not shown). Moreover, the Ewens-Watterson’s F test of neutrality [40, 41] for all HLA loci did not show a deviation from expectations (*p* = 0.8) (Additional file 2: Table S9).

### Evolutionary mechanisms inferred from mismatch distribution and Bayesian skyline plot

**Fig. 3:**
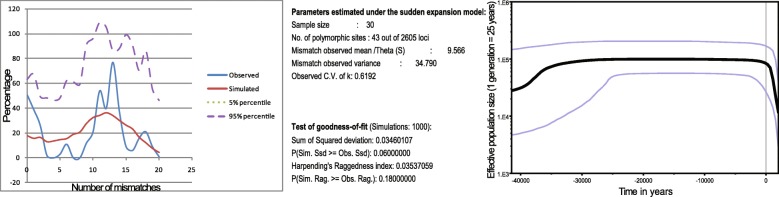
Mismatch distribution analysis (MMA) and Bayesian Skyline Plot (BSP) obtained from mtDNA nps 8000–9000, 10,000–11,000 and HVS-I. MMA: the hypothesis of sudden expansion is not rejected by the SSD test (*P* = 0.06) [42]. BSP [45]: From an expanded population of ~ 3600 women, the Thao effective population today is approximately 400 and agrees with a recent survey of 660 Thao males and females [1]

A finite-sites mutation model for mtDNA nps 8000-9000, 10,000-11,000, and 16,040–16,400 with empirical 95% confidence intervals was used to determine the mismatch distribution in Thao (Fig. 3, left) [42, 43]. As expected in equilibrium populations, the coefficient of variation of the average pairwise differences was large (CV = 0.62). Further, the sum of the square deviation test (SSD test; *P* = 0.06) did not reject the hypothesis of sudden expansion and was further confirmed by the Fu’s Fs neutrality tests (Fu’s Fs = − 24.34527, *p* < 0.001) [44]. Because of the low number of Thao individuals used in the analysis, the Bayesian skyline plot (Fig. 3, right) did not reveal much evolutionary structure [45], and results should be interpreted with caution. As it stands, the demographic curve first suggested a long period of population stability before reaching a sudden decline in the effective population size during the last two millennia. This may support alarming historical events during which the Thao people must have gone through considerable periods of relocation, hardship, and adaptation to new environments [17].

### Multiple dimensional scaling (MDS) and putative parental contribution analysis

**Fig. 4:**
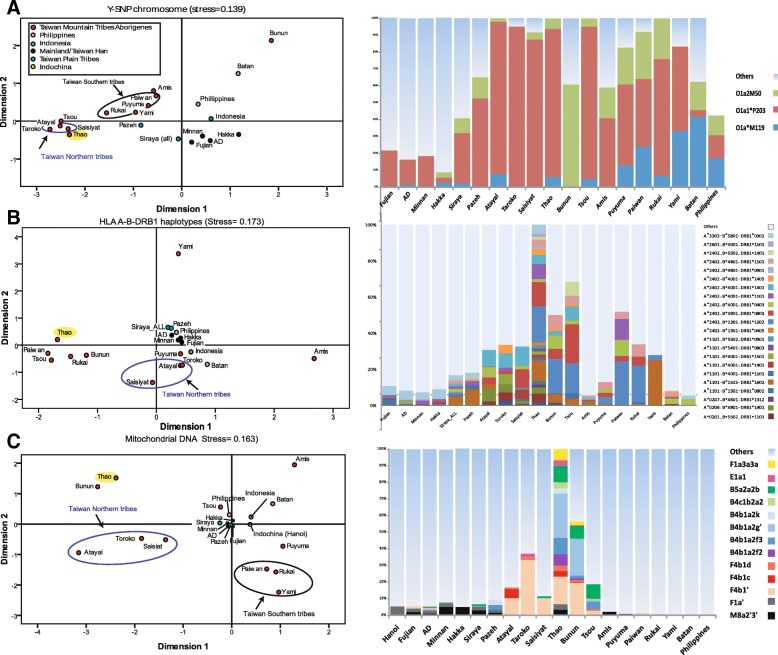
Thao haplogroup sharing distribution (right) and Multiple dimensional scaling plots (MDS, left) constructed based on Fst distances using haplogroup/haplotype frequencies distribution for three gene systems (**a**: Y-SNP, **b**: HLA-A-B-DRB1, and **c**: mtDNA) and relevant populations data from the literature [27, 31, 34, 36]. In each MDS plot, Thao is highlighted in yellow and colors characterizing other groups are described in the insert of “A”. Blue and black circles surrounding population groups indicate northern and southern groups of Taiwan recognized indigenous peoples. On the right, the light blue color above the bar-plots (labeled “others” on the right) represents polymorphism not seen in Thao. Grey colors represent non-Taiwan Aboriginal admixture. Although scarce in Fujian, the mtDNA haplogroup F4b1’ is considered to be a Taiwan indigenous peoples characteristic

Multiple dimensional scaling plots representing genetic affinity between Taiwan groups are shown in Fig. 4 (Fig. 4, left, Y-SNP, HLA-A-B-DRB1 haplotypes, and mtDNA respectively). We first note the outlying position of the Bunun in the Y-SNP MDS corresponding to their low diversity and the unexpectedly high frequency of O1a2-M50 and O2a1a (Additional file 1: Table S1) [27]. This is most likely the result of early male-specific gene flow from southeastern mainland Asia or from west-coast plains peoples (Taiwan Pingpu) followed by a bottleneck, founder effect, and drift after isolation of the Bunun in the central mountain range. Second, the three MDS plots revealed greater genetic differentiation among the groups. The Thao people were invariably associated with the northern and central TwrIP (Atayal, Taroko, Saisiyat, Tsou, and Bunun), clearly separated from the TwPp, the Han (Fujian, Minnan, Hakka, and TwMx), and the peoples of Philippines and Indonesia.

After having established a definite ancestral affinity between the Thao and the northern and central TwrIP, we looked at the genetic distribution of the three gene systems, HLA, mtDNA, and Y-chromosome (Fig. 4 right, and Additional file 1: Table S1). The Y-chromosome SNP profile of Thao showed higher affinity with Atayal and Tsou than with Fujian or non-TwA. Most interesting was the very close mtDNA affinity seen between Thao and Bunun, likely attributable to the confined distribution of the B4b1a2 subclades among the northern and central mountain peoples (Additional file 3: Supplementary text 1), a finding also supported by Blust on linguistic grounds [16]. In sum, with the exception of the HLA affinity of the Thao with the southern Paiwan and Rukai peoples, the Y-chromosome and mtDNA profiles substantiate the HLA profile in characterizing the Thao as a member of the northern/central mountain peoples.

### Contribution analysis

Two putative parental groups were used in Table 4 to infer the genetic makeup of the Thao, a parental group representing the Han (Fujian), and an Austronesian-speaking group comprised of a pool of all Taiwan indigenous peoples but Thao. Parental contribution [46] was calculated according to Y-SNP, 7 Y-STR, HLA-A-B-DRB1 and mtDNA gene families (Table 3). The Y-STR analysis indicated greater Han contribution to Thao (43%) than when using only Y-SNP (25%). Actually, inspection of the O1a1a*P203 Y-STR haplotypes Network (cluster 2 in Fig. 2) indicated that 9 out of 13 unshared Y-STR in the Thao cluster where identical and the cluster represented a male isolation period of 1590 YBP (Table 2). Most likely, three factors, a restricted Y-chromosome sample size, low genetic diversity, and rapid drift may have contributed to this difference. However, the results shown above suggest that the Thao have a Neolithic ancestry similar to other recognized indigenous peoples of Taiwan [47, 48].

**Table 4:** Gene contribution to Thao from two putative parent groups

Putative Parental populations	Thao			
	HLA-A*-B*-DRB1*	Y-SNP	Y-STR	mtDNA
Taiwan officially recognized Indigenous people not including Thao (TwrIP)	55.2%	75.0%	57.0%	75.3%
Fujian (Han)	44.8%	25.0%	43.0%	24.7%

Note: The sum of parental contribution sums to one (Maca-Meyer et al. 2004)

## Discussion

It is generally believed that the Taiwan Pingpu groups (such as Pazeh and Siraya) were initially Austronesian speakers who belonged to the same group of people as the Taiwan mountain peoples today [17] (Fig. 1 and Additional file 8: Figure S1). According to archeological and linguistic evidence, they arrived in Taiwan during the early Neolithic from Southeast China approximately 6000 years ago [49]. As the result of continuous and numerous arrivals from China, largely Minnan and Hakka, in the last 400 years, the Neolithic settlers who remained in the more hospitable environment of the western plains of Taiwan are presently heavily culturally and genetically Sinicized [25, 31, 34, 35]. Knowledge of the genetic boundaries between Taiwan aborigines and Taiwan Han is important in reconstructing the heritage of these groups in relation to ancient and modern events, and for the design and implementation of genetic epidemiologic studies.

The Thao Aborigines today are a small and sinicized indigenous group in central Taiwan. Because of their language, the Thao peoples have been classified as a plains people [50]. Their language actually neared extinction in the past few hundred years as the number of individuals fell to approximately 260, and their language in 2000 was then only competently spoken by less than 15 Thao individuals [15, 16]. The official recognition by the Taiwan government in 2001 of the Thao as an indigenous people contributed to the revival and preservation of their ethnic cultures and language. Presently, their language contains loan words from the Bunun ethnic group with whom they mixed and intermarried [16]. More interestingly, the presence, in the Thao language, of specific cognates allows retracing their ancestry to Proto-Austronesian groups [16]. However, debates on their ethnic status and origin are ongoing.

Herein we used genetic information obtained from mtDNA, HLA-A-B-DRB1, 16 Y-STRs, and 81 Y-SNPs to shed light on their origin.

First, Multi-Dimensional-scaling (MDS) analyses, using the three gene systems (Fig. 4) invariably grouped the Thao among the mountain peoples. Moreover, MDS showed a strong paternal influence from the northern peoples, Atayal, Saisiyat, and Taroko, and a strong maternal affinity of Thao with the central peoples, Bunun and Tsou.

The high level of cultural Sinicization of the Thao during the last four centuries is contrasted by the observed lower than expected level of Han genetic admixture for mtDNA and Y chromosome (24.5 to 44.8% respectively).

This mtDNA admixture result was well supported by the evolutionary mechanisms of the Thao inferred from Mismatch Distribution which produced a multimodal curve indicating a past period of female introduction into the Thao. However, according to Harpending [42, 43] an mtDNA diversity as low the one seen in the Thao (Additional file 1: Table S1) and a multimodal curve of the mismatch distribution (Harpending raggedness = 0.035) (Fig. 3, left) possibly indicate an ancestral period with few founding genes, rapid drift, or most likely, admixture events.

The lower HLA-A-B-DRB1 haplotype diversity in Thao (0.939) than in non-Taiwan aborigines (0.995) and Han (0.997) (Additional file 1: Table S1 and Additional file 9: Table S8) suggested that, despite modernization and the strong Han influence of the last 400 years, the Thao have managed to conserve their genetic heritage. The MDS plots (Fig. 4) clearly reflect the important role of the physical impact played by the central mountain ranges in isolating the Thao from later Han gene flow and for the conservation of the original Thao genetic profiles that are seen across the three gene systems used in this study.

Previous contacts with the ancestors of the Pazeh plains people proposed by linguistic researchers [15] were not refuted by our results. The sharing of genetic traits between the Thao and Pazeh could only have happened at a very early stage during the settlement of the Austronesian agriculturists in the western plain of Taiwan. At that time, the plains peoples and mountain peoples had not yet separated and had sprung from the same southeastern Mainland Asian gene pool, and Y-SNP haplogroups O1a1*P203 and mtDNA haplogroup B4b1a2 were just beginning to diversify from their ancestral founding branches [3, 29] (Additional file 8: Figure S1). The predominance in Thao of specific gene types such as B4b1a2g’f’k and F1b1’c’d, may be the result of later female gene flow from other recognized central mountain peoples (Bunun and Tsou) introduced after the Thao had left the western plain [11, 15–17] (Additional file 1: Table S1).

For the male counterpart, haplogroup O1a1*P203 in the Thao (87.5%) produced a unique Y-STR network showing no sharing of Y-STRs haplotypes with other Formosan groups, and having an age estimate of molecular variation of 1590 ± 690 YBP (Table 2, Fig. 2 and Additional file 1: Table S1). It is possible that this low age estimate is the consequence of a male bottleneck following bad health or the result of the very small number of Thao survivors forced to relocate several times during the last few centuries [17]. This unique genetic structure further suggests that a small homogeneous group of males, bearers of O1a1*P203 and having strong bonding to their patriarchal culture, managed to remain untouched by male external gene flow in the last two millennia. Any contact with the ancestors of the Pazeh could only have happened before that period. Through maintaining their traditions (Shamanism, patrilineality, the Ulalaluan symbol of ancestry, folktales, and most importantly, their plains tribal language), the Thao have succeeded in conserving a cultural heritage which characterizes them as a discrete member of the other Formosan groups [11, 15–17]. In retracing their physical journey from the western plains to the central mountain range, we showed that the Thao also succeeded in preserving a Formosan genetic signature which is one that is highly likely to have been shared by all the plains and mountain peoples of the early Neolithic, before the arrival of Han settlers and genetic Sinicization (Additional file 8: Figure S1).

## Conclusions

This study has exploited the advantages of using multiple highly polymorphic gene systems as an efficient method to supplement often restricted uniparental chromosome analysis and to deliver robust support to previous genetic, anthropological, archaeological and linguistic studies, linking proto-Austronesians with the Neolithic cultures of Taiwan. At the same time, rapid progress in complete genome sequencing is opening new avenues in population analysis, in particular for disease analyses. The success of this growing field is largely dependent on the availability of data obtained from groups with high homozygosity or out of neutrality equilibrium. This situation presents special problems to the research scientists, as the unique genetic structure of the Taiwan aboriginal peoples and other once isolated aboriginal groups are rapidly being modified through dispersal, social interactions, acculturation, and admixture. Many genetic disease association studies would greatly benefit from the analysis of small aboriginal groups and vice versa. This source of important human genetic data has yet to be systematically used. Without urgent action, their genetic data will be lost forever. Despite the shortcomings introduced in this study by the small number of Thao individuals used, we show that a small aboriginal group, under strong admixture pressure, successfully conserved its ancestral genetic structure, and we raise the awareness of the urgency to create a methodology for exploring the genetic structure of other rare population groups.

## Material and methods

### Population samples

The Thao genetic diversity for Y-chromosome, mtDNA, and HLA was determined in 30 unrelated (back to two generations) and healthy individuals. All individuals had both parents and first-generation grandparents belonging to the same people and gave consent to participate in this study. Approval to conduct this project was obtained from the ethics committee of Mackay Memorial Hospital in Taipei (Taiwan).

The Thao data set (Additional file 9: Table S8) was compared to a panel of other Taiwan individuals that we had previously analyzed for Y-chromosome [27], mtDNA [31, 33] and HLA. The HLA data is available online at http://www.allelefrequencies.net and in the proceedings of the Anthropology/HLA diversity component of the 13th international histocompatibility workshop [24, 25, 34, 51, 52]. Geographic locations and sampling sites of the Taiwanese groups used for a comparative purpose are shown in Fig. 1. This panel comprises a) a dataset of non-Taiwan aborigines that includes Minnan (*n* = 672), Hakka (*n* = 200) and a sample of undefined number of Minnan and Hakka, referred to herein as TwMix (*n* = 3227), b) Taiwan officially recognized indigenous peoples (TwrIP) including Atayal (*n* = 110), Taroko or Truku (*n* = 54), Saisiyat (*n* = 64), Bunun (*n* = 181), Tsou (*n* = 60), Rukai (*n* = 78), Paiwan (*n* = 172), Amis (*n* = 294), Puyuma (*n* = 116) Yami/Tao (*n* = 88), Ivatan/Batan (*n* = 50), and c) indigenous Taiwan Pingpu peoples (TwPp, *n* = 493) including Pazeh (*n* = 65) and Siraya groups (*n* = 428). To obtain a more detailed analysis, we selected other in-house material: Eastern Chinese (Fujian, *n* = 149, Philippines, *n* = 317, and Batan n = 50) [31, 33, 53, 54]. Phylogenetic analysis was improved through the use of additional data from the literature, principally complete-mtDNA genome typing from Phylotree [3, 6, 55] and NRY Y-STR [26, 48] (Additional file 10: Table S6).

### Preparation and sequencing

Genomic DNA was extracted from 500 μl of buffy coat using the QIAamp DNA Blood Mini Kit (Qiagen inc. Chatsworth, California, United States) with minor adjustments to the procedure recommended by the manufacturer.

Mitochondrial haplogroup assignments were obtained by comparing known reference genomes [55] to the nucleotide variation of the D-loop HVS-I control region (nucleotide positions nps 16,006–16,397) and coding regions (nps 8000–9000, nps 9959–10,917 and nps 14,000–15,000) according to our previously published sequencing protocol [31]. Ambiguous haplogroup assignments were confirmed using further pertinent sequencing of segments of the coding region [31, 56, 57].

Complete mitochondrial genome sequencing for this study was obtained for each representative haplotype of the Thao people using our previously published sequencing protocol [31].

Y-Chromosome polymorphism was determined using 81 NRY markers, the majority of which are slowly evolving binary markers (Y-SNPs), according to published sequencing protocols [27, 56]. In brief, sequencing was performed on both strands using the DiDeoxy Terminator Cycle Sequencing Kit (Applied Biosystems) according to manufacturer recommendations. Purification on a G50 Sephadex column was performed before the final run on an automated DNA Sequencer (ABI Model 377). The nomenclature used for haplogroup labeling is in agreement with the classification provided by the International Society of Genetic Genealogy for the Y Chromosome Consortium and recent updates [56, 58].

Further genotyping with of 16 microsatellites markers (DYS19, DYS385I, DYS385II, DYS389II, DYSS390, DYS391, DYS392, DYS393, DYS437, DYS438, DYS439, DYS448, DYS456, DYS458, DYS635, and Y GATA-H4) was done using the Y-filter kit (Applied Biosystems) following the manufacturer’s instructions. In brief, PCR products were mixed with GeneScan 500LIZ (Applied Biosystems) as an internal size standard and analyzed by capillary electrophoresis with an ABI Prism 310 genetic analyzer (Applied Biosystems) using the standard fragment analysis protocol mode. Genotyper 2.5.2 software (Applied Biosystems) was used for allele scoring. For all statistical and network analyses, we used data from DYS389II by subtracting DYS389I from DYS389II [29].

### Statistical analyses

The Thao frequencies of haplogroups of the Y-SNP and mtDNA gene systems, and of the HLA-A, −B and -DB1 alleles were obtained by mere counting (Additional file 9: Table S8). The HLA-A-B-DRB1 haplotype data were estimated using the EM algorithm in Arlequin version 3.5.2.2 (Additional file 1: Table S1 and Additional file 9: Table S8). To validate these frequencies in the Thao, the linkage disequilibrium of each haplotype was inferred and goodness of fit was calculated using the Pearson’s cumulative chi-squared test statistic χ 2 (Additional file 9: Table S8). [59, 60]. The unbiased gene diversity index, *h*, and its standard error were calculated using the formulas given by Nei [61] (Additional file 8: Figure S1). Molecular diversity, Tajima D: [62], Fu’s Fs [44], mismatch difference analysis (MMDA) [42], and pairwise population distances (*F*_*ST*_) [63] were calculated using Arlequin version 3.1143 [59]. Demographic variation through time was obtained from a Bayesian skyline plot (BSP) [45] using Beast with a relaxed molecular clock and a mutation rate of 2.2964 × 10^− 7^ mutations per site per year for the mtDNA HVS1 data (Fig. 3).

Y-STR Median-Joining (MJ) networks restricted to a single Y-SNP haplogroup were constructed using Network v. 4.5.1.6 (Fluxus Engineering; http://www.fluxus-engineering.com) after processing the data with the reduced-median method and weighting the STR loci proportionally to the inverse of the repeat variance (Fig. 2). The age of Y microsatellite variation was obtained using the rho statistic method of Zhivotovsky et al. [28] and modified according to Sengupta et al. [64] (Table 2). Haplogroups age estimates for mtDNA were calculated from the complete genome variation rate of one substitution every 3624 years using the rho statistic [65] and corrected for purifying selection as implemented by Soares [4] (Table 3). Dates were only intended as a rough guide for relative haplogroup ages comparison. Multiple Dimension Scaling Analysis plots (MDS) using haplogroup frequencies of the three gene systems (Fig. 4) were constructed with SPSS version 17.01 using Alscal Euclidian distances (SPSS Inc., Chicago IL).

MtDNA HVS1 region and complete mtDNA sequencing described herein have been deposited in GenBank (GenBank sequence submission of 38 complete mtDNA genome, MH177784- MH177821). Y-chromosome STR data and partial mtDNA sequencing are provided in Additional file 10: Table S6 and Additional file 11: Table S7. Other NRY Y-STR and Y-SNP data sets are available on [27].

## Additional files

Additional file 1: **Table S1.** Frequencies of all gene systems. (XLS 546 kb)

Additional file 2: **Table S9.** Neutrality tests in all populations. (XLSX 10 kb)

Additional file 3: **Supplementary text 1.** Genetic diversity of the Thao tribe of Taiwan using Y-Chromosome, mitochondrial DNA and HLA gene systems. Recovery from near extinction. (DOCX 81 kb)

Additional file 4: **Table S2.** B4b1a2 phylogenetic tree. (XLS 502 kb)

Additional file 5: **Table S3.** B5a2a phylogenetic tree. (XLS 502 kb)

Additional file 6: **Table S4.** F1a3 phylogenetic tree. (XLS 502 kb)

Additional file 7: **Table S5.** F4b phylogenetic tree. (XLS 494 kb)

Additional file 8: **Figure S1.** Proposed model for simulation of migration and admixture. AN: Austronesian speakers; NRY: Non-recombining Y chromosome. 1. End of Pleistocene (before 15,000 YBP: O3 is primarily seen in Eastern China, O1 in SEA and O2 in Indochina). 2. Local Expansion of mtDNA and NRY haplogroups into subtypes (examples: O1 to O1a*M119, O1a1*P203 and O1a2-M50). 3. Austronesian speakers in Taiwan (Between 6000 and 4000 YBP). Aborigines plains peoples in the western plains and mountain peoples share the same NRY and mtDNA gene pools. Most carry NRY haplogroup O1a1*P203. 1. First Mainland gene flow with the introduction of NRY O3, A, C, and other Y haplogroups. At that time the Thao were a plains people beginning their migration towards the central mountain range. 2. Thao complete male isolation, and mtDNA sharing (with Bunun and Tsou) until the present days. 3. 400 YBP, Chinese migration to Taiwan. 4. The Taiwan plains peoples have been heavily sinicized. Through successive relocations, the Thao escaped contact from mainland gene flow. The Thao people represent the last plains people who successfully conserved their Austronesian culture and ancestral genome. They only recently emerged from extinction and are now expanding in the area around Sun Moon Lake in the central Taiwan mountain range). (PDF 350 kb)

Additional file 9: **Table S8.** Thao HLA-A*, B* and DRB1* alleles, haplotype linkage, and three loci haplotype frequencies in all populations. (XLSX 183 kb)

Additional file 10: **Table S6.** Thao NRY SNPs and STRs. (XLS 525 kb)

Additional file 11: **Table S7.** Partial and complete mtDNA genome(raw data). (XLSX 92 kb)

## Abbreviations

BSP: Bayesian Skyline plot
HLA: Histoleucocyte antigens
MDS: Multiple dimensional scaling
mtDNA: Mitochondrial DNA
Non-TwA: Non Taiwan Aborigines
np(s): nucleotide position(s)
NRY: Non recombining Y chromosome
PCR: Polymerase chain reaction
SNP: Single-Nucleotide Polymorphism
TwPp: Taiwan Pingpu
TwrIP: Taiwan recognized indigenous peoples
YBP: Year before present
Y-STR: Y chromosome single strand repeat
