# Correction to: Genetic diversity of the Thao people of Taiwan using Y-chromosome, mitochondrial DNA and HLA gene systems

**DOI:** 10.1186/s12862-019-1540-y

**Published:** 2019-11-20

**Authors:** Jean A. Trejaut, Frank Muyard, Ying-Hui Lai, Lan-Rong Chen, Zong-Sian Chen, Jun-Hun Loo, Jin-Yuan Huang, Marie Lin

**Affiliations:** 1 Molecular Anthropology and Transfusion Medicine Research Laboratory, Mackay Memorial Hospital, Taipei, Taiwan; 2 Department of French Studies, National Central University, Taoyuan Taiwan & French School of Asian Studies (EFEO), Taoyuan, Taiwan

**Correction to: BMC Evol Biol (2019) 19:64**

**https://doi.org/10.1186/s12862-019-1389-0**

Following publication of the original article [1], we have been notified that Additional file 3 was published with track changes.

In this correction, the revised supplementary material is included.

## Supplementary information

**Additional file 3: Supplementary text 1.** Genetic diversity of the Thao tribe of Taiwan using Y-Chromosome, mitochondrial DNA and HLA gene systems. Recovery from near extinction.
