# Supplementary material for: Genetic diversity of the Thao people of Taiwan using Y-chromosome, mitochondrial DNA and HLA gene systems
Source: BMC Evol Biol. 2019 Feb 27;19:64. doi: 10.1186/s12862-019-1389-0 (PMC6391829; doi:10.1186/s12862-019-1389-0)
Supplement: Supplementary file 3 — Supplementary text 1. Genetic diversity of the Thao tribe of Taiwan using Y-Chromosome, mitochondrial DNA and HLA gene systems. Recovery from near extinction. (DOCX 81 kb) [file 12862_2019_1389_MOESM3_ESM.docx]

**Supplementary Text 1**

**Title: Genetic diversity of the Thao people of Taiwan using Y-chromosome, mitochondrial DNA and HLA gene systems. Recovery from near extinction.**

Jean A. Trejaut^1^*, Muyard, Frank^2^,Ying-Hui Lai^1^, Lan-Rong Chen^1^, Zong-Sian Chen^1^, Jun-Hun Loo^1^, Jin-Yuan Huang^1^ and Marie Lin^1^*

^1^-Molecular Anthropology and Transfusion Medicine Research Laboratory, Mackay Memorial Hospital, Taipei, Taiwan

^2^-Department of French Studies, National Central University, Taoyuan, Taiwan, & French School of Asian Studies (EFEO), Taoyuan, Taiwan

**Authors' email addresses:**

Trejaut, Jean Alain jtrejaut@gmail.com

### Lai, Ying-Hui [ptkc20002000@yahoo.com.tw](mailto:ptkc20002000@yahoo.com.tw)

Lan-Rong, Chen [lchen@livemail.tw](mailto:lchen@livemail.tw)Loo, Jun-Hun junhun@ms1.mmh.org.tw

### Chen, Zong-Sian zxchen@ ms1.mmh.org.tw

### Huang, Jin-Yuan sammuhuang108@gmail.com

### Lin, Marie marilin@mmh.org.tw

### Muyard, Frank frank.muyard@gmail.com

**Address for corresponding authors:**

Email: [marilin@ms2.mmh.org.tw](mailto:marilin@ms2.mmh.org.tw) or jtrejaut@gmail.com

***Haplogroups B4b1a2in Thao***

The well-structured distribution of haplogroup B4b1a2(np 6216)[[1](#_ENREF_1)] (Additional file 4: Table S2) suggests gene flow continuity between mainland Asia and ISEA, but no sharing of the most recent lineages are seen between distant regions. Members of the founding B4b1a clade have been identified in mainland Southeast Asia (Indochina) and Japan (as B4a1a1 or B4b1a2a respectively), and Siberia (as B4b1a3)[[2](#_ENREF_2)]. At a more refined assignment level, twigs of B4b1a2 have been seen at low frequency among the Taiwan Han [[3-5](#_ENREF_3)], in Tuvalu, Guadalcanal, the Solomon Islands [[6](#_ENREF_6)], and even as a single occurrence in a Peruvian Chechuan [[7](#_ENREF_7)]. Further, the subtypes of B4b1a2 seen among mainland Chinese are notably distinct from those seen among TwrIP (3 subtypes) or Filipinos (12 other subtypes distinct from Taiwan and China) [[4](#_ENREF_4), [5](#_ENREF_5)] (Supplementary Table S2). This disparate distribution suggests a multidirectional dispersal of the founding haplogroup B4b1a2 from SEA approximately 9,314 ya (CI 6,958-11,032 ya)[[1](#_ENREF_1)] (Table 2b). Along with the dispersal of rice-agriculturists to Taiwan, bearers of B4b1a2 reached Northeast Asia (NEA) and expanded locally (Additional file 4: Table S2). In Taiwan, subclades of B4b1a2 (named f, g and, k in Additional file 4: Table S2) are principally seen among central TwrIP groups (Thao, Bunun, Tsou), and Amis on the East Coast. The convergence age estimate of the three subclades (4200 to 4900 ya, Table 3) suggests that these subclades most likely remained isolated among the TwrIP to the present day. A similar pattern, with limited expansion of other subtypes of B4b1a2, is seen in the Philippines and supports a common genetic signal of the Neolithic expansion for Taiwan and the Philippines as suggested by Brandao and others [[5](#_ENREF_5), [6](#_ENREF_6), [8](#_ENREF_8)]. Interestingly, the dispersal of B4a1a2 to Taiwan and ISEA shows strong similarities to the expansion of M7c3cand its later connection to the Out of Taiwan (OOT) model [[5](#_ENREF_5), [9](#_ENREF_9)].

In Thao, B4b1a2f3 (np G709A, T14110C and A6527G), B4b1a2g (np C16365T) and B4b1a2k (nps 207!, 8014 and 16400) represents approximately 47% of the population. The diversity of most mtDNA haplogroups, more specifically of the B4b1a2 clade, is lower in Thao than in Bunun (Additional file 1: Table S1 and Additional file 4: Table S2). This suggests that the Thao profile is the result of drift and gene flow from the Bunun tribe. We note that the presence of B4b1a2in Tsou could indicate alternative contributing gene flow to the Thao female community.

Intriguingly, findings of individuals with B4b1a2k (np G207A!) have been reported in Pazeh (this study), an Alzheimer’s patient from Japan [[2](#_ENREF_2)], Negritos, and non-Negritos from the Philippines [[5](#_ENREF_5), [8](#_ENREF_8), [10](#_ENREF_10)]. The large spread of this dispersal, the poor phylogenic power of nucleotide position G207A! for the haplogroup assignation B4b1a2k, and the additional presence of npsA8014G and C16400T in the lineages seen in Thao and Pazeh, may justify a review of the phylogeny of B4b1a2k and make a B4b1a2k gene flow in Thao from the Philippines or Japan unlikely.

In summary, the localized distribution and the high diversity of the B4b1a2 clade seen in central TwrIP (Bunun, Thao, Tsou, and Amis), the Philippines, and the absence of B4b1a2 gene flow between Taiwan and the Philippines in the last 5000 years,suggests a wide initial Neolithic dispersal of the founding haplogroup B4b1a2 from SEA to Northeast Asia,Taiwan, and the Philippines in the last 7500 years BP, followed by separate local speciations when contact between Pazeh and Thao was still a possibility (Additional file 8: Figure S1). A signal for this dispersal was also recently proposed by Lipson et al. (2014), although they favored admixture with Austronesian agriculturists dispersing around the coasts of MSEA as an explanation, which our results render unlikely.

***Haplogroup B5a2***.

B5a2 is believed to be a postglacial haplogroup having found its origin in mainland South China. It is prevalent among the Hmong-Mien groups [[11](#_ENREF_11)] and it links its bearers with the Neolithic cultures of these regions, such as the Daxi Culture (5,300-6,400 YBP) and the Qujialing Culture (4,600-5,000 YBP) [[11](#_ENREF_11)]. While B5a2a1 is most seen in China, B5a2a2, a subclade rooted by npT8614C, has been seen in Japan [[1](#_ENREF_1)] (Additional file 5: Table S3), and rarely in the Philippines or Indonesia [[5](#_ENREF_5), [9](#_ENREF_9)]. In Taiwan, B5a2a2a (npsA15046G and T16362C) is common among TwrIP, scarce among the Pingpu (Additional file 1: Table S1 and Additional file 5: S3), and prevalent among the southern TwrIP Paiwan and Rukai. Conversely, B5a2a2b (nps C5027T and C8059T), with a molecular variation estimate of 6228 ya (CI 2760-9772) is more commonly seen in the central TwrIP, like the Bunun (7.4%), Tsou (8.3%), and Thao (10%).

**Haplogroup F1a3**

**F1a3** has been reported in Taiwan, the Philippines and Indonesia [[3](#_ENREF_3), [12-14](#_ENREF_12)] and is rarely seen among mainland Chinese or Taiwan Han groups (Additional file 1: Table S1 and Additional file 6: Table S4). While its origin remains unclear, its founding lineages most likely expanded in southern and eastern China [[15](#_ENREF_15)] during the late Paleolithic era. Haplogroup F1a3a (G9554A, T9944C, A13748G) must have reached Japan, the Philippines, and south Taiwan in the early Holocene to pre-Neolithic (Table 3) along with Neolithic farmers. In Taiwan, this is confirmed with the presence of the F1a3a3 clade (np C15452T; 4951 YBP, CI 954-9052) and several of its twigs among the Puyuma, Tsou, and Bunun. The presence of F1a3a3 also in Pingpu (Syraya), possibly because some Bunun recently migrated to Pingtung county in southern Taiwan. Interestingly, two F1a3a subtypes, most likely of Taiwan origin (F1a3a2, F1a3a3) reached Tao island (Yami) and the Batan archipelago in the north of the Philippines (Loo). These subtypes should perhaps be considered as temporary members of the OOT gene pool [[5](#_ENREF_5)]. Finally, the Thao subtype F1a3a3a (G14323A; ~2377 ya, CI 97-4692) appears to reflect a molecular expansion that occurred only among Thao and Bunun and suggests close contact between the two tribes. A few subtypes of F1a3, not previously described, have been included in Additional file 6: Figure S4 with tentative assignments.
Last, F1a5 ( npsG6962A T10604C C14043T) most likely arrived in Taiwan along with the F1a3 clade. It is seen principally among non-Taiwan aborigines except for a single sub-lineage (nps G3736A and A14053G) in the Thao.

**Haplogroup F4b**

**F4b** has been extensively described by Brandao [[5](#_ENREF_5)]. Its presence in the Thai, Vietnamese, and southern China (Additional file 7: Table S5) suggests an early Holocene dispersal from Southeast Asia (6897 ya CI 1068-12939) with branches expanding over China (F4b1 and F4b2) and Indochina (F4b3) (Additional file 7: Table S5). It is sparse in south China, Hainan, and the Taiwan Han [[3-5](#_ENREF_3), [16](#_ENREF_16)]. With a coalescence age estimate of 4317 ya (CI 1296-7280), F4b1 (np T10097C) is the type most commonly seen among TwrIP. It is rare in the Philippines [[14](#_ENREF_14)]. Except for F4b1b (A16241G) found in Japan and the non-aboriginal peoples of Taiwan, most branches of F4b1 are confined to the northern and central TwrIP (Atayal, Toroko, Saisiyat, Bunun, Thao, Tsou, and Amis)[[3](#_ENREF_3), [4](#_ENREF_4)]. Together, F4b1c’d constitute the second most prevalent types among the Thao (23%) (Additional file 1: Table S1, Additional file 7: Table S5, and Fig.4).

Lastly, haplogroups B4c1b2a2 (T195C and G15301A), M8a2, and E1a1 were identified only once in the Thao. Variants of B4c1b2a2 have been seen in the Philippines, Orchid Island, western ISEA, Malaysia [[5](#_ENREF_5)], and more rarely, in the non-aboriginal populations of Taiwan and Fujian. Further, we note that the E1a1 lineage is a new type whose phylogeny appears to be close to the sequence of Liangdao Man [[4](#_ENREF_4)], differing only at nps 4248-6620-10834 and 16129 (data not shown). M8a2 may be the result of modern gene flow.

**References**

1. van Oven M, Kayser M: **Updated comprehensive phylogenetic tree of global human mitochondrial DNA variation**. *Hum Mutat* 2009, **30**(2):E386-394.

2. Tanaka M, Cabrera VM, Gonzalez AM, Larruga JM, Takeyasu T, Fuku N, Guo LJ, Hirose R, Fujita Y, Kurata M *et al*: **Mitochondrial genome variation in eastern Asia and the peopling of Japan**. *Genome Res* 2004, **14**(10A):1832-1850.

3. Trejaut JA, Kivisild T, Loo JH, Lee CL, He CL, Hsu CJ, Lee ZY, Li ZY, Lin M: **Traces of archaic mitochondrial lineages persist in Austronesian-speaking Formosan populations**. *PLoS Biol* 2005, **3**(8).

4. Ko AM, Chen CY, Fu Q, Delfin F, Li M, Chiu HL, Stoneking M, Ko YC: **Early Austronesians: into and out of Taiwan**. *Am J Hum Genet* 2014, **94**(3):426-436.

5. Brandao A, Eng KK, Rito T, Cavadas B, Bulbeck D, Gandini F, Pala M, Mormina M, Hudson B, White J *et al*: **Quantifying the legacy of the Chinese Neolithic on the maternal genetic heritage of Taiwan and Island Southeast Asia**. *Hum Genet* 2016, **135**(4):363-376.

6. Duggan AT, Evans B, Friedlaender FR, Friedlaender JS, Koki G, Merriwether DA, Kayser M, Stoneking M: **Maternal history of Oceania from complete mtDNA genomes: contrasting ancient diversity with recent homogenization due to the Austronesian expansion**. *Am J Hum Genet* 2014, **94**(5):721-733.

7. Achilli A, Perego UA, Bravi CM, Coble MD, Kong QP, Woodward SR, Salas A, Torroni A, Bandelt HJ: **The phylogeny of the four pan-American MtDNA haplogroups: implications for evolutionary and disease studies**. *PLoS One* 2008, **3**(3):e1764.

8. Delfin F, Min-Shan Ko A, Li M, Gunnarsdottir ED, Tabbada KA, Salvador JM, Calacal GC, Sagum MS, Datar FA, Padilla SG *et al*: **Complete mtDNA genomes of Filipino ethnolinguistic groups: a melting pot of recent and ancient lineages in the Asia-Pacific region**. *Eur J Hum Genet* 2014, **22**(2):228-237.

9. Soares PA, Trejaut JA, Rito T, Cavadas B, Hill C, Eng KK, Mormina M, Brandao A, Fraser RM, Wang TY *et al*: **Resolving the ancestry of Austronesian-speaking populations**. *Hum Genet* 2016, **135**(3):309-326.

10. Gunnarsdottir ED, Li M, Bauchet M, Finstermeier K, Stoneking M: **High-throughput sequencing of complete human mtDNA genomes from the Philippines**. *Genome Res* 2011, **21**(1):1-11.

11. Lee GY: **Diaspora and the Predicament of Origins: Interrogating Hmong Postcolonial History and Identity**. *Hmong Studies Journal* 2007( 8):1-25.

12. Hill C, Soares P, Mormina M, Macaulay V, Clarke D, Blumbach PB, Vizuete-Forster M, Forster P, Bulbeck D, Oppenheimer S *et al*: **A mitochondrial stratigraphy for island southeast Asia**. *Am J Hum Genet* 2007, **80**(1):29-43.

13. Scholes C, Siddle K, Ducourneau A, Crivellaro F, Jarve M, Rootsi S, Bellatti M, Tabbada K, Mormina M, Reidla M *et al*: **Genetic diversity and evidence for population admixture in Batak Negritos from Palawan**. *Am J Phys Anthropol* 2011, **146**(1):62-72.

14. Tabbada KA, Trejaut J, Loo JH, Chen YM, Lin M, Mirazon-Lahr M, Kivisild T, De Ungria MC: **Philippine mitochondrial DNA diversity: a populated viaduct between Taiwan and Indonesia?***Mol Biol Evol* 2010, **27**(1):21-31.

15. Zheng HX, Yan S, Qin ZD, Wang Y, Tan JZ, Li H, Jin L: **Major population expansion of East Asians began before neolithic time: evidence of mtDNA genomes**. *PLoS One* 2011, **6**(10):e25835.

16. Peng MS, He JD, Liu HX, Zhang YP: **Tracing the legacy of the early Hainan Islanders--a perspective from mitochondrial DNA**. *BMC Evol Biol* 2011, **11**:46.
