# Supplementary figures and images for: Genetic diversity of the Thao people of Taiwan using Y-chromosome, mitochondrial DNA and HLA gene systems
Source: BMC Evol Biol. 2019 Feb 27;19:64. doi: 10.1186/s12862-019-1389-0 (PMC6391829; doi:10.1186/s12862-019-1389-0)

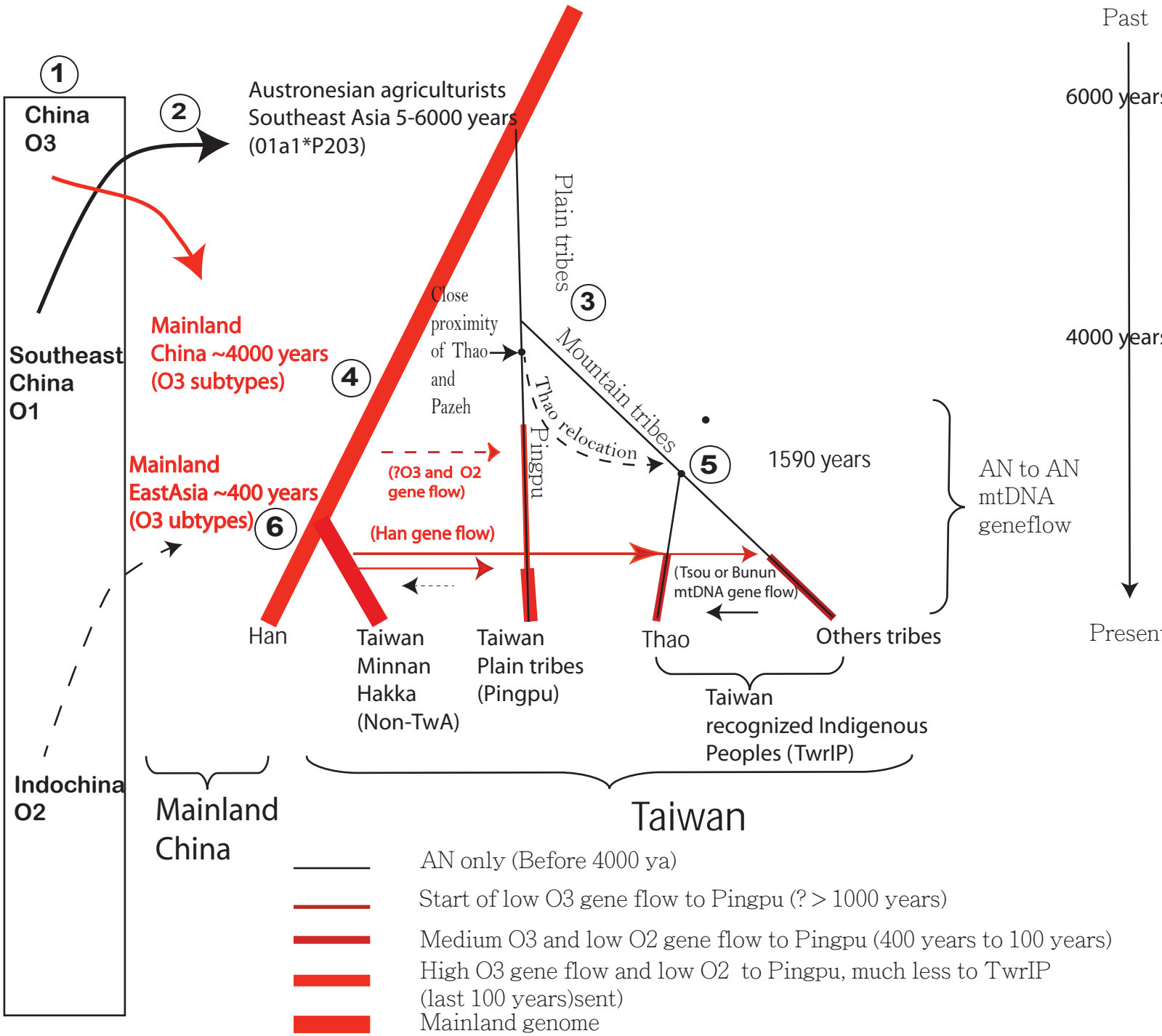

Supplement: Supplementary file 8 — Figure S1. Proposed model for simulation of migration and admixture. AN: Austronesian speakers; NRY: Non-recombining Y chromosome. 1. End of Pleistocene (before 15,000 YBP: O3 is primarily seen in Eastern China, O1 in SEA and O2 in Indochina). 2. Local Expansion of mtDNA and NRY haplogroups into subtypes (examples: O1 to O1a*M119, O1a1*P203 and O1a2-M50). 3. Austronesian speakers in Taiwan (Between 6000 and 4000 YBP). Aborigines plains peoples in the western plains and mountain peoples share the same NRY and mtDNA gene pools. Most carry NRY haplogroup O1a1*P203. 1. First Mainland gene flow with the introduction of NRY O3, A, C, and other Y haplogroups. At that time the Thao were a plains people beginning their migration towards the central mountain range. 2. Thao complete male isolation, and mtDNA sharing (with Bunun and Tsou) until the present days. 3. 400 YBP, Chinese migration to Taiwan. 4. The Taiwan plains peoples have been heavily sinicized. Through successive relocations, the Thao escaped contact from mainland gene flow. The Thao people represent the last plains people who successfully conserved their Austronesian culture and ancestral genome. They only recently emerged from extinction and are now expanding in the area around Sun Moon Lake in the central Taiwan mountain range). (PDF 350 kb) [file 12862_2019_1389_MOESM8_ESM.pdf]
